# Antioxidant Activity of Mulberry Fruit Extracts

**DOI:** 10.3390/ijms13022472

**Published:** 2012-02-22

**Authors:** Muhammad Arfan, Rasool Khan, Anna Rybarczyk, Ryszard Amarowicz

**Affiliations:** 1 Department of Chemistry, Palosa Campus, Abdul Wali Khan University, 24420 Charsadda, KPK, Pakistan; E-Mails: m_arfan@upesh.edu.pk (M.A.); rasoolkhan1@hotmail.com (R.K.); 2 Institute of Animal Reproduction and Food Research of the Polish Academy of Sciences, Tuwima Street 10, 10-747 Olsztyn, Poland; E-Mail: a.rybarczyk@pan.olsztyn.pl

**Keywords:** mulberry, *Morus nigra*, *Morus alba*, antioxidants, phenolic compounds

## Abstract

Phenolic compounds were extracted from the fruits of *Morus nigra* and *Morus alba* using methanol and acetone. The sugar-free extracts (SFEs) were prepared using Amberlite XAD-16 column chromatography. All of the SFEs exhibited antioxidant potential as determined by ABTS (0.75–1.25 mmol Trolox/g), DPPH (2,2-diphenyl-1-picrylhydrazyl) (EC_50_ from 48 μg/mL to 79 μg/mL), and reducing power assays. However, a stronger activity was noted for the SFEs obtained from *Morus nigra* fruits. These extracts also possessed the highest contents of total phenolics: 164 mg/g (methanolic SFE) and 173 mg/g (acetonic SFE). The presence of phenolic acids and flavonoids in the extracts was confirmed using HPLC method and chlorogenic acid and rutin were found as the dominant phenolic constituents in the SFEs.

## 1. Introduction

The mulberry belongs to the *Morus* genus of the *Moraceae* family. There are 24 species of *Morus*, with at least 100 known varieties [1]. Mulberry leaves, bark and branches have long been used in Chinese medicine [2]. In most European countries mulberries are grown for fruit production [3,4].

Plants of the genus *Morus* are known to be a rich source of flavonoids including quercetin 3-(-malonylglucoside), rutin, isoquercitin [5], cyanidin 3 rutinoside and cyanidin 3-glucoside [6,7]. A high content of total phenolics in white, red, and black mulberry fruits was reported by Ercisli and Orhan [8]. Using capillary electrophoresis with amperometric detection, Chu *et al*. [9] identified apigenin, luteolin, quercetin, morin, caffeic acid, gallic acid, rutin, umbelliferone, chlorogenic acid, and kaempferol in the fruit of *Morus alba*.

The biological activity of *Mallotus* leaves and fruit extracts or individual chemical constituents isolated from these extracts have been reported. The flavonol glycosides isolated from mulberry leaves [quercetin 3-(-malonylglucoside), rutin, and isoquercitin] inhibited human LDL oxidation induced by copper [5]. Mulberry leaf powder prevented atherosclerosis in apolipoprotein E-deficient mice [10]: the group fed a diet containing 1% of mulberry leaf showed a 40% reduction in atherosclerotic lesion size in the aortae compared with the control. Using the inhibition test, the methanolic extract of Indian mulberry leaves inhibited the anti-tumour-promoting activity of Epstein-Barr virus [11], and the phytoestrogens in a *Morus rubra* extract appeared to be active at specific developmental stages [12].

The antioxidant potential of the extracts obtained from mulberry leaves and fruits was investigated by several authors. Among the 28 fruits commonly consumed in China, mulberry pulp was characterised by one of the highest values of the ferric reducing antioxidant power (FRAP) at 4.11 mmol/100 g wet weight [13]. The antioxidant properties of mulberry leaf extracts were investigated by Arabshahi-Delouee and Urooj [14] by various experimental methods including the iron (III) reducing capacity, the total antioxidant capacity, the DPPH (2,2-diphenyl-1-picrylhydrazyl) radical scavenging activity and an *in vitro* inhibition of ferrous sulphate-induced oxidation of lipids. The percentage of superoxide ion scavenged by extracts of mulberry leaves, mulberry tender leaves, mulberry branches and mulberry bark were 46.5, 55, 67.5 and 85.5%, respectively, at a concentration of 5 μg/mL [2]. The antioxidant activities of ethanolic extracts of five mulberry cultivars from Korea were determined using the DPPH radical assay, haemoglobin-induced linoleic acid system, and reducing power. The extracts were found to be strong scavengers of hydroxyl radicals and superoxide anions [15].

To the best of our knowledge, there have been no comparative studies on the antioxidant potential of the *Morus alba* and *Morus nigra*, grown under the same climatic condition. Therefore, the aim of this work was to obtain extracts from white and black mulberry species, and assess their relative antioxidant activities.

## 2. Results and Discussion

Polyphenolic compounds are some of the most effective antioxidative constituents in plant foods such as fruits, vegetables, and grains; thus it is important to quantify their polyphenolic contents and to assess their contribution to antioxidant activity. The total polyphenolic contents (TPC) of the sugar-free extracts (SFEs) of mulberry fruits were expressed as mg catechin equivalents per g. The highest TPC was obtained for a *Morus nigra* acetonic SFE (173 mg/g), followed by a *Morus nigra* methanolic SFE (164 mg/g); the lowest TPC was obtained for a *Morus alba* methanolic SFE (119 mg/g) (Table 1). In this study, the crude extracts were subjected to column chromatography to remove the sugars. Therefore, the TPC of our extracts (SFEs) was much higher than crude extracts containing sugars that are obtained from such plant material as apples [16], legumes [17], nuts [18], herbs [19], and cereals [20]. Using extracts obtained with 70% ethanol for 4 h at room temperature, the crude extract of five different Korean cultivars of *Morus alba* fruits showed only 0.95–2.57 mg gallic acid equivalents (GAE)/g [15]. In the study of Ericisli and Orhan [3] the content of total phenolics of *Morus nigra* fruits (1422 mg GAE/100 g fresh mass) similar to our work was higher than for *Morus alba* (181 mg GAE/100 g fresh mass). However, the opposite results were reported by Imran *et al.* [21], namely that the contents of total phenolics in mulberry fruits were 6.64 mg/100 g fresh mass (*Morus nigra*) and 7.55 mg/100 g fresh mass (*Morus alba*). Arabshahi-Delouee and Urooj [14] reported that mulberry leaf extracts obtained using methanol, acetone and water exhibited 93, 85, and 71 mg total phenolics/g, respectively.

In our study, the total antioxidant activity of the SFEs ranged from 0.75 mmol Trolox/g (*Morus alba* methanolic SFE) to 1.25 mmol Trolox/g (*Morus nigra* methanolic SFE). In general, the SFEs from the *Morus nigra* fruits exhibited greater total antioxidant activities than those from the *Morus alba* fruits. The antiradical activity of a mulberry crude extract against the ABTS radical cation was reported previously by Tsai *et al*. [22].

**Figure 1:**
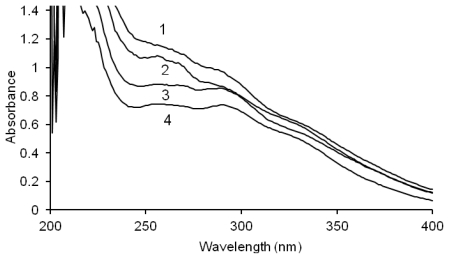
UV spectra of mulberry fruits sugar-free extracts (SFEs); (**1**) *Morus nigra* methanolic SFE, (**2**) *Morus nigra* acetonic SFE; (**3**) *Morus alba* methanolic SFE, (**4**) *Morus alba* acetonic SFE.

The UV spectra of the phenolic compounds present in the SFEs are provided in Figure 1. The spectra of SFEs of the *Morus alba* fruits were characterised by maxima that were better separated. The absorption bands at wavelengths of 320–350 nm confirm the presence of phenolic acids and most probably flavonoids in the extracts.

**Figure 2:**
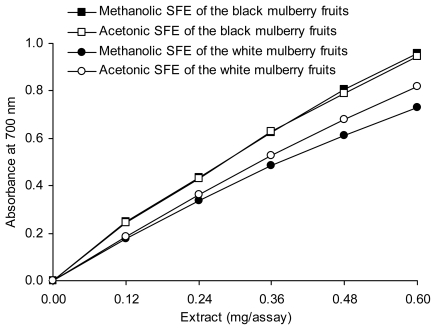
Reducing power of mulberry fruits SFEs as measured by changes in absorbance at 700 nm.

Figure 2 shows the reducing power of the SFEs. The results indicate that the acetonic and methanolic SFEs of *Morus nigra* exhibited almost the same reducing power, which was stronger than those observed for the SFEs of *Morus alba.* A similar reducing power of the methanolic extract of *Morus indica* leaves was reported by Arabshahi-Delouee and Urooj [14]. The ability of mulberry extract to reduce Fe^3+^ to Fe^2+^ was reported by Tsai *et al*. [22], Iqbal *et al*. [23], and Koca *et al.* [24] using the FRAP assay.

**Figure 3:**
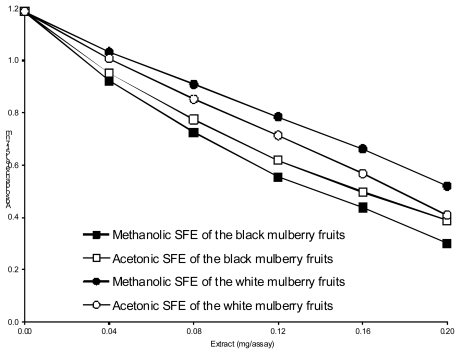
Antiradical activity of mulberry fruits SFEs against DPPH radical as measured by changes in absorbance at 517 nm.

Figure 3 depicts the dose-response curves for the DPPH radical-scavenging activity of the mulberry SFEs. The results indicate that acetonic and methanolic SFEs of *Morus nigra* were more active scavengers than the SFEs of *Morus alba.* The SFEs exhibited values of EC_50_ from 48 (methanolic SFE of *Morus nigra*) to 79 μg/mL (methanolic SFE of *Morus alba*). The result obtained in this study is high, whereas much weaker abilities to scavenge DPPH radicals by the extracts of *Morus nigra* and *alba* fruits was observed by Imran *et al.* [21]. The antiradical activity against the DPPH radical was reported by Zhang *et al*. [25] for anthocyanins (cyanidin-3-glucoside and cyanidin-3-rutinoside) isolated from *Morus alba* polmace.

The HPLC chromatograms of the SFEs recorded at 320 and 360 nm were characterised by the presence of six dominant peaks (1–6) with a retention time of 6.7, 11.9, 13.6, 13.9, 18.5, and 37.8 min, respectively. The DAD-UV spectra of the compounds producing peaks 1–3 exhibited maximum at 324 nm, the spectra of compounds 4 and 5 were characterised by maxima at 354 nm, and the spectrum of compound 6 possessed a maximum at 349 nm. Using original standards, compound 2 was identified as a chlorogenic acid and compound 4 as rutin. The contents of both above mentioned compounds in SFEs of *Morus nigra* were higher than those in the SFEs of *Morus alba* (Table 2). However, the highest content of compound 5 was found in the SFEs of *Morus alba*. In the study of Chu *et al.* [9] chlorogenic acid was the dominant phenolic acid identified in mulberry fruits, and the authors also reported the presence of such flavonoids as rutin, quercetin, kaempferol, morin, apigenin, and luteolin.

## 3. Experimental Section

The fruits of *Morus alba* and *Morus nigra* were collected from the Buner area in Pakistan. Unless otherwise specified, all solvents used for the chromatography were of HPLC grade.

Ferric chloride, potassium ferricyanide, potasium persulfate, trifluoroacetic acid (TFA) and trichloroacetic acid (TCA) were acquired from the P.O.Ch. Company (Gliwice, Poland). (+)- Catechin, chlorogenic acid, rutin, Folin and Ciocalteu’s phenol reagent, 2,2-diphenyl-1-picrylhydrazyl radical (DPPH·), 2,2′-azinobis-(3-ethylbenzothiazoline-6-sulfonic acid) (ABTS), and 6-hydroxy-2,5,7,8-tetramethylchroman-2-carboxylic acid (Trolox) were purchased from Sigma Ltd. (Poznań, Poland).

The phenolic compounds were extracted from mulberry fruits using 80% (v/v) acetone or methanol 80% (v/v) at a solid to solvent ratio of 1: 10 (w/v), at 50 °C for 30 min [26]. The extraction was carried out in dark-colored flasks using a shaking water bath (Elpan 357, Wrocław, Poland). The extraction was repeated twice more, the supernatants were combined and the acetone was evaporated under vacuum at 40 °C in a rotary evaporator (Unipan 359P, Poznań, Poland); the remaining aqueous solution was lyophilised.

The sugars present in the crude extract of the mulberry fruits were removed using column chromatography [27]. The extract was applied on the column (40 × 2 cm) packed with Amberlite XAD-16 resin washed with water, and then sugars were eluted from the column with 1 L of water. The phenolic compounds remaining in the column were then eluted with 400 mL methanol and concentrated under vacuum.

The UV spectra of the extracts dissolved in methanol were recorded using a Beckman DU 7500 diode array spectrophotometer (Beckman Instruments, Fullerton, CA, USA).

The content of total phenolics in the extracts was determined according to the procedure described by Naczk and Shahidi [28] using the Folin-Ciocalteu’s reagent. (+)-Catechin was used as a standard in this work.

The total antioxidant capacity in the extracts was determined according to the Trolox equivalent antioxidant activity (TEAC) assay described by Re *et al*. [29] and was expressed as mmol Trolox equivalent/g of the extract.

The reducing power of the extracts was determined by the method of Oyaizu [30]. Briefly, the assay medium contained 2.5 mL of the sample extract in 0.2 M phosphate buffer (pH 6.6) and 2.5 mL of 1% (w/v) potassium ferricyanide. After incubation at 50 °C for 20 min, 2.5 mL of 10% (w/v) trichloroacetic acid was added to the mixture followed by centrifugation at 1750 × *g* for 10 min. The supernatant (2.5 mL) was mixed with 2.5 mL distilled water and 0.5 mL of 0.1% (w/v) ferric chloride, and the absorbance of the resulting solution was measured at 700 nm.

The capacity of the prepared extracts to scavenge the “stable” free radical 2,2-diphenyl-1-picrylhydrazyl (DPPH·) was monitored according the procedure described by Amarowicz *et al*. [20]. Briefly, 0.1 mL of methanolic solution containing 0.04 to 0.20 mg of extract was mixed with 2 mL of distilled water and then 0.25 mL of 1 mM methanolic solution of the DPPH radical was added. The mixture was vortexed thoroughly for 1 min. Finally, the absorbance of the mixture was measured at 517 nm after standing at ambient temperature for 30 min.

The phenolic constituents of the extracts were analysed using a Shimadzu HPLC system (Shimadzu Corp., Kyoto, Japan) consisting of two LC-10AD pumps, an SCTL 10A system controller and an SPD-M 10A photodiode array detector. The chromatography was carried out using a pre-packed LiChrospher 100 RP-18 column (4 × 250 mm, 5 μm; Merck, Darmstadt, Germany). Elution for 50 min in a gradient system of 5–40% acetonitrile in water adjusted to pH 2.5 with TFA was employed [31]; injection volume was 20 μL and the flow rate was 1 mL/min.

All analytical determinations in this study were triplicated.

## 4. Conclusions

The data presented in this report demonstrate that the sugar-free extracts of *Morus nigra* and *alba* have great antioxidant potential, as determined by ABTS, DPPH, and reducing power assays. We suggest that they may be used as alternatives to synthetic antioxidants.

## Figures and Tables

**Table 1:** Characteristics of mulberry fruit sugar-free extracts (SFEs).

Cultivar	Extraction solvent	Total phenolics (mg/g)	Total antioxidant activity (mmol Trolox/g)	EC_50_* (μg/mL)
*Morus nigra*	Methanol	164 ± 5 ^a^	1.25 ± 0.06 ^a^	48 ± 1 ^a^
	Acetone	173 ± 4 ^a^	1.19 ± 0.04 ^a^	58 ± 1 ^b^
*Morus alba*	Methanol	119 ± 3 ^b^	0.75 ± 0.01 ^b^	79 ± 1 ^c^
	Acetone	140 ± 5 ^b^	0.78 ± 0.02 ^b^	66 ± 1 ^d^

* The concentration of SFE that reduces one-half of the initial amount of DPPH radical in the experimental sample. The data are expressed as the means ± standard deviations; values of the same cultivar and extraction solvent having different letters differ significantly (*P* < 0.05).

**Table 2:** Content of individual phenolic compounds in SFEs of mulberry fruits (mg/g).

Compound	Extraction solvent	*Morus nigra*	*Morus alba*
1	Methanol	10.8 ± 0.6 ^a^	7.4 ± 0.3 ^b^
	Acetone	12.1 ± 0.6 ^a^	10.2 ± 0.5 ^b^
2 (chlorogenic acid)	Methanol	21.3 ± 1.55 ^a^	15.0 ± 0.7 ^b^
	Acetone	23.3 ± 1.1 ^a^	17.7 ± 0.8 ^b^
3	Methanol	14.5 ± 0.63 ^a^	10.2 ± 0.52 ^b^
	Acetone	15.5 ± 0.7 ^a^	12.7 ± 0.6 ^b^
4 (rutin)	Methanol	39.7 ± 2.12 ^a^	26.2 ± 1.2 ^b^
	Acetone	43.0 ± 2.2 ^a^	32.3 ± 1.6 ^b^
5	Methanol	15.1 ± 0.67 ^a^	28.5 ± 1.4 ^b^
	Acetone	17.3 ± 0.7 ^a^	34.3 ± 1.6 ^b^
6	Methanol	12.3 ± 0.6 ^a^	14.0 ± 0.7 ^b^
	Acetone	14.7 ± 0.6 ^a^	16.5 ± 0.6 ^b^

The results are expressed as the means ± standard deviations; the values of the same cultivar and extraction solvent having different letters differ significantly (*P* < 0.05). Compounds 1 and 2 were expressed as chlorogenic acid, compounds 5 and 6 as rutin.
